# Sixteen years post radiotherapy of nasopharyngeal carcinoma elicited multi-dysfunction along PTX and chronic kidney disease with microcytic anemia

**DOI:** 10.1186/1471-2490-14-19

**Published:** 2014-02-12

**Authors:** Yi-Ting Lin, Chia-Chun Huang, Charng-Cherng Chyau, Kuan-Chou Chen, Robert Y Peng

**Affiliations:** 1 Department of Urology, St. Joseph’s Hospital, 74, Sinsheng Road, Huwei County, Yunlin Hsien 632, Taiwan; 2 Research Institute of Biotechnology, Hungkuang University, 34 Chung-Chie Road, Shalu County, Taichung City 43302, Taiwan; 3 Department of Radiation Oncology, Changhua Christian Hospital, No.135 Nan Shiau Street, Changhua 500, Taiwan; 4 Department of Urology, Taipei Medical University-Shuang Ho Hospital, Taipei Medical University, 250, Wu-Xin St, Xin-Yi District 110 Taipei, Taiwan; 5 Department of Urology, School of Medicine, Taipei Medical University, 250, Wu-Xin St, Xin-Yi District 110 Taipei, Taiwan

**Keywords:** Radiotherapy, Hypopituitarism, Hypothyroidism, Hypogonadism, Adrenal insufficiency, Chronic kidney disease, Microcytic anemia

## Abstract

**Background:**

The hypothalamic–pituitary (h-p) unit is a particularly radiosensitive region in the central nervous system. As a consequence, radiation-induced irreversible, progressively chronic onset hypopituitarism (RIH) commonly develops after radiation treatments and can result in variably impaired pituitary function, which is frequently associated with increased morbidity and mortality.

**Case presentation:**

A 38-year-old male subject, previously having received radiotherapy for treatment of nasopharygeal carcinoma (NPCA) 16 years ago, appeared at OPD complaining about his failure in penile erection, loss of pubic hair, atrophy of external genitalia: testicles reduced to 2×1.5 cm; penile size shrunk to only 4 cm long. Characteristically, he showed extremely lowered human growth hormone, (HGH, 0.115 ng/mL), testosterone (<0.1 ng/mL), total thyroxine (tT4: 4.740 g/mL), free T4 (fT4, 0.410 ng/mL), cortisol (2.34 g/dL); lowered LH (1.37 mIU/mL) and estradiol (22 pg/mL); highly elevated TSH (7.12 IU/mL). As contrast, he had low end normal ACTH, FSH, total T3, free T3, and estriol; high end normal prolactin (11.71 ng/mL), distinctly implicating hypopituitarism-induced hypothyroidism and hypogonadism. serologically, he showed severely lowered Hb (10.6 g/dL), HCT (32.7%), MCV (77.6 fL), MCH (25.3 pg), MCHC (32.6 g/dL), and platelet count (139×103/L) with extraordinarily elevated RDW (18.2%), together with severely lowered ferritin (23.6 ng/mL) and serum iron levels; highly elevated total iron binding capacity (TIBC, 509 g/dL) and transferrin (363.4 mg/dL), suggesting microcytic anemia. Severely reduced estimated glomerular filtration rate (e-GFR) (89 mL/mim/1.73 m2) pointed to CKD2. Hypocortisolemia with hyponatremia indicated secondary adrenal insufficiency. Replacement therapy using androgen, cortisol, and Ringer’s solution has shown beneficial in improving life quality.

**Conclusions:**

To our believe, we are the first group who report such complicate PTX dysfunction with adrenal cortisol insufficiency concomitantly occurring in a single patient.

## Background

The hypothalamic–pituitary (h-p) unit is a particularly radiosensitive region of the central nervous system. As a consequence, radiation-induced hypopituitarism (RIH) commonly develops after radiation treatments [1,2]. RIH, an irreversible and progressive chronic onset disorder, can result in a variable impairment of pituitary function [1,2], usually associated with increased morbidity and mortality [3].

Selective radiosensitivity of the neuroendocrine axes, with the human growth hormone (HGH) axis being the most vulnerable, accounts for the high frequency of HGH deficiency (GHD) following irradiation of the h–p axis with doses less than 30 Gy [2]. Life table analysis shows that the percent damages are dose- and time-dependent, and the frequency of GHD can substantially increase to reach as high as 50–100% [3]. At average, to lose 75% of the normal axis function of GHD may take 3.3 years, for LH/FSH, 7.8 years; and ACTH, 8.2 years [4]. Abnormalities in gonadotrophin secretion occurs dose-dependently but infrequently, and hyperprolactinemia is usually subclinical [2].

Thyroid hormones, directly or indirectly through erythropoietin, stimulate growth of erythroid colonies. Anemia is often the first sign of hypothyroidism diagnosed in 20-60% patients with hypothyroidism [5]. Numerous mechanisms are involved in the pathogenesis of these anemias which can be microcytic, macrocytic and normocytic [6]. Microcytic anemia is usually ascribed to malabsorption and failure in transport of iron. Macrocytosis is found in up to 55% patients with hypothyroidism and may result from the insufficiency of the thyroid hormones themselves without nutritive [5] like vitamin B_12_ and folic acid, and is frequently seen in pernicious anemia. Normocytic anemia is characterized by reticulopenia, hypoplasia of erythroid lineage, and decreased level of erythropoietin and mainly is associated with regular erythrocyte survival [6]. Worth note, pernicious anemia occurs 20 times more frequently in patients with hypothyroidism than generally [5].

We report a male patient who showed apparently shrunken penis and reduced testicular size, erectile dysfunction and loss of libido 16 years post radiotherapy of his nasopharyngeal carcinoma (NPCA).

## Case presentation

**Figure 1:**
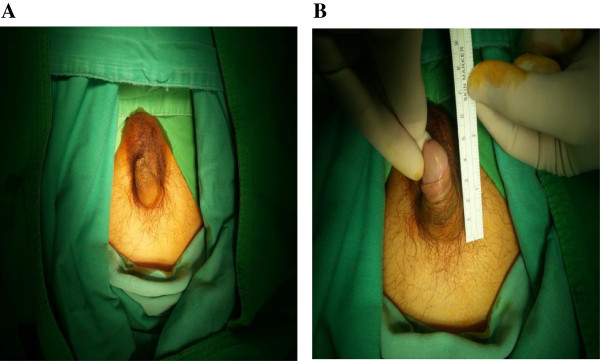
**Appearance of this genital organ-penis.** Shrunken penis is apparently with size reduced to only 4 cm long post radiotherapy for treating nasopharyngeal carcinoma (NPCA) 16 years ago. (**A**: Penis regularly appearing, **B**: Measuring size of penis)(patient: male, age 38).

A 38 years old male patient visited the Urological Outpatient Department and complained mainly about complete loss of penile erection already for half a year. The fact was soon recognized that he had been diagnosed to be with NPC, epidermoid carcinoma, nonkeratinizing in July 2, 1997, Stage cT3N2bM0 (previous AJCC staging system, 4^th^ edition, not AJCC 7^th^ staging system). The overall cumulative radiotherapy dose was 7140 cGy/42 fractions. The fraction dose was 170 cGy. The irradiation was delivered with conventional 2-dimensional radiotherapy using the linear accelerator equipped with 6 MV energy. Complete tumor response was achieved. The physical examination revealed shrunken external genitalia with penile size severely reduced to only 4 cm long accompanied with complete loss of pubic hair (Figure 1). His testicles are apparently with atrophy, size severely reduced to approximately 2×1.5 cm (not shown). Tracing back to the past history, the patient was diagnosed with nasopharyngeal carcinoma (NPCA) and as in the above mentioned he had received cumulative radiotherapy dose 7140 cGy/42 fractions (170 cGy/per fraction) sixteen years ago and didn’t receive any extra surgery or chemotherapy for this NPCA. The NPCA was successfully treated and didn’t recur up to present. In the same duration, he successfully went on to father three healthy children, age 16, 15 and 10 respectively.

**Figure 2:**
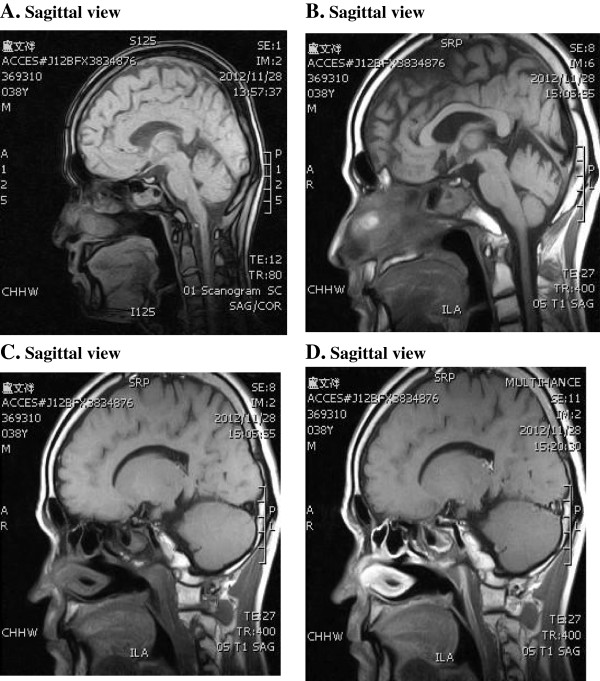
**MRI scanogram of patient Lu’s brain.** The sagittal views (pictures **A-D**) reveal the hypophysis has been injured severely and the nasal pharyngeal area is severely deformed.

**Figure 3:**
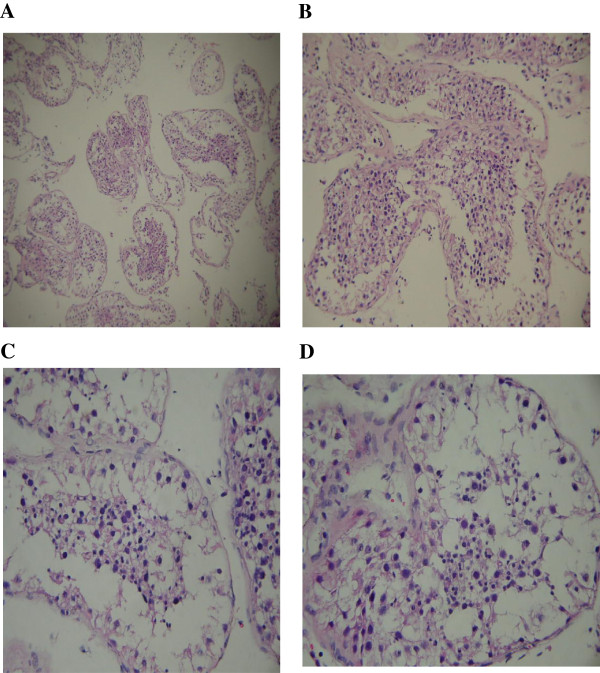
**Hematoxylin-Eosin stain of testicualr tissues. A**: 100×; **B**: 200×; **C**: 300×; and **D**: 400× (date: December 20, 2012). The thickening of seminiferous tubular walls is a typical symptom of early testicular atrophy. The Leydig cells and Sertoli cells appear normal with mature spermatogenesis, however with reduced density and lack of compact cell linings as usually seen in normal subjects.

The sagittal views reveal his hypophysis has been injured severely. The nasal pharyngeal area is severely deformed. (Figure 2). After testis biopsy, the pathological report showed pictures of mature testis with spermatogenesis, well developed seminiferous tubules and Leydig cells, yet the number of mature spermatozoa is fair per cross section of seminiferous tubules. The thickening of tubular wall indicates early stage testicular atrophy (Figure 3).

The laboratory data revealed extremely lowered HGH, lower levels of LH, testosterone, E2, total thyroxine and free T4, accompanied with severely lowered cortisol, low end level ACTH, FSH, total T3, and free T3; highly elevated TSH, high end normal prolactin, and normal estriol were also noticed (Table 1). Complete blood count revealed low RBC, severely lowered Hb, HCT, MCV, MCH, MCHC, PLT, MPV, elevated RDW (Table 2); severely lowered ferritin, low serum iron levels, highly elevated serum total iron binding capacity, elevated transferrin and the low end marginal e-GFR (Table 3). This patient was commenced on replacement therapy with cortisone injections, Ringer’s solution (3%), thyroxine (T4), and testosterone replacement were immediately prescribed.

**Table 1:** Endocrine analysis

**Hormone**	**Value**	**Normal range**	**Comment**
ACTH, pg/mL	12.2	9.0–52.0 (website 1)	Low end normal
GH, ng/mL	0.115	1.000–9.000 (website 2)	Severely lower
Prolactin, ng/mL (EIA)	11.71	male: 2.64–13.13; 4–23 (website 3); (3–15, website 4)	High end normal
FSH, mIU/mL (EIA/LIA)	4.91	males: 1.27–19.26; 1.4–18.1 (male) (website 5)	Low end normal
LH, mIU/mL	1.37	males: 1.50–9.30 (website 5)	Lower
Estradiol, E2 (EIA/LIA), pg/mL	22	males: 25–70 (website: 4)	Lower
Estriol (E3) (EIA), pg/mL	<0.017	0–3 (website: 6)	Normal
Testosterone (EIA/LIA), ng/mL	<0.1	Blood: 3.0–12.0 (website 5)	Trace only
T4 total (biochem), μg/dL	4.740	6.09–12.23	Extremely lower
Free T4 (EIA/LIA), ng/dL	0.41	0.54–1.40	Extremely lower
T3 total, (EIA/LIA), ng/dL	92.88	87.00–178.00	Low end normal
Free T3 (EIA/LIA), pg/mL	2.780	2.500–3.900; 2.300–6.190 (website 7)	Low end normal
TSH (EIA/LIA), μIU/mL	7.12	0.34–5.60	Highly elevated
Cortisol, μg/dL	-	-	-
At 8 am	2.34	6.7–22.6	Severely lowered;
At 16 pm	3.74	0–10	Low end

Websites: 1. http://www.nlm.nih.gov/medlineplus/ency/article/003695.htm.

2. Medline Plus. 3. Selleckchem.com. 4-30 ng/ml in females and 4-23 ng/mL in males. 4. MedHelp. 5. Health Board. 6. High-Pro.com. 7. Men’s Health Message Board. 7. About.Com. Thyroid disease. Practice Notebook. Updated July 31, 2003; by Kenneth N. Woliner, M.D., A.B.F.P. Website: Nova Tech ImmunodiagnosticaGmbH.

**Table 2:** Complete blood count*

	**Value obtained**	**Normal range**	**Comment**
WBC, 10^3^/μL	5.2	3.5–9.6	Normal
RBC, 10^6^/μL	4.21	4.20–6.23	Low end margin
Hb, g/dL	10.6	13–18	Severely lower
HCT, %	32.7	38.8–53.1	Severely lower
MCV, fL	77.6	80.0–100.0	Severely lower
MCH, pg	25.3	27.0–32.0	Severely lower
MCHC, g/dL	32.6	33.3–36.0	Lower
PLT, 10^3^/μL	139	150–450	Severely lower
PDW, %	17.6	15.5–18.0	Normal
MPV, fL	6.6	6.8–13.5	Lower
RDW, %	18.2	12.1–15.2	Highly elevated

^*^WBC: white blood cells. RBC: red blood cells. Hb: hemoglobin. HCT: hematocrit. MCV: mean corpusular volume. MCH: mean corpusulr hemoglobin. MCHC: mean corpusular hemoglobin concentration. PLT: platelet count. PDW: platelet distribution width. MPV: mean platelet volume. RDW: red blood cell distribution width.

**Table 3:** Biochemical tests of serum

**Parameters**	**Value obtained**	**Normal range**	**Comment**
Serum BUN, mg/dL	16	8–20	Normal
s-GOT (AST), IU/L	28	15–41	Normal
s-GPT (ALT), IU/L	14	10–40	Normal
TIBC, μg/dL	509	255–450	Highly elevated
Ferritin (EIA), ng/mL	23.6	30.0–336.2	Severely lower
Transferrin (Nephelometry), mg/dL	363.4	180–329	Highly elevated
Serum Iron (Fe), μg/dL	55	Adult male: 45–182	Lower margin
Creatinine (bood), mg/dL	0.95	0.70–1.20	Normal
e-GFR, mL/min/1.73 m^2^	89	90–120	Low end margin
Na^+^, mEq/L or mmol/L	124	135–146	Severely lower
Cu^2+^ (blood), μg/dL	129.75	70-140	Normal

TIBC: Total iron binding capacity. e-GFR: estimated glomerular filtration rate.

s-GOT: serum glutamate-oxaloacetate transaminase. s-GPT: serum glutamate-pyruvate transaminase.

## Discussion

**Figure 4:**
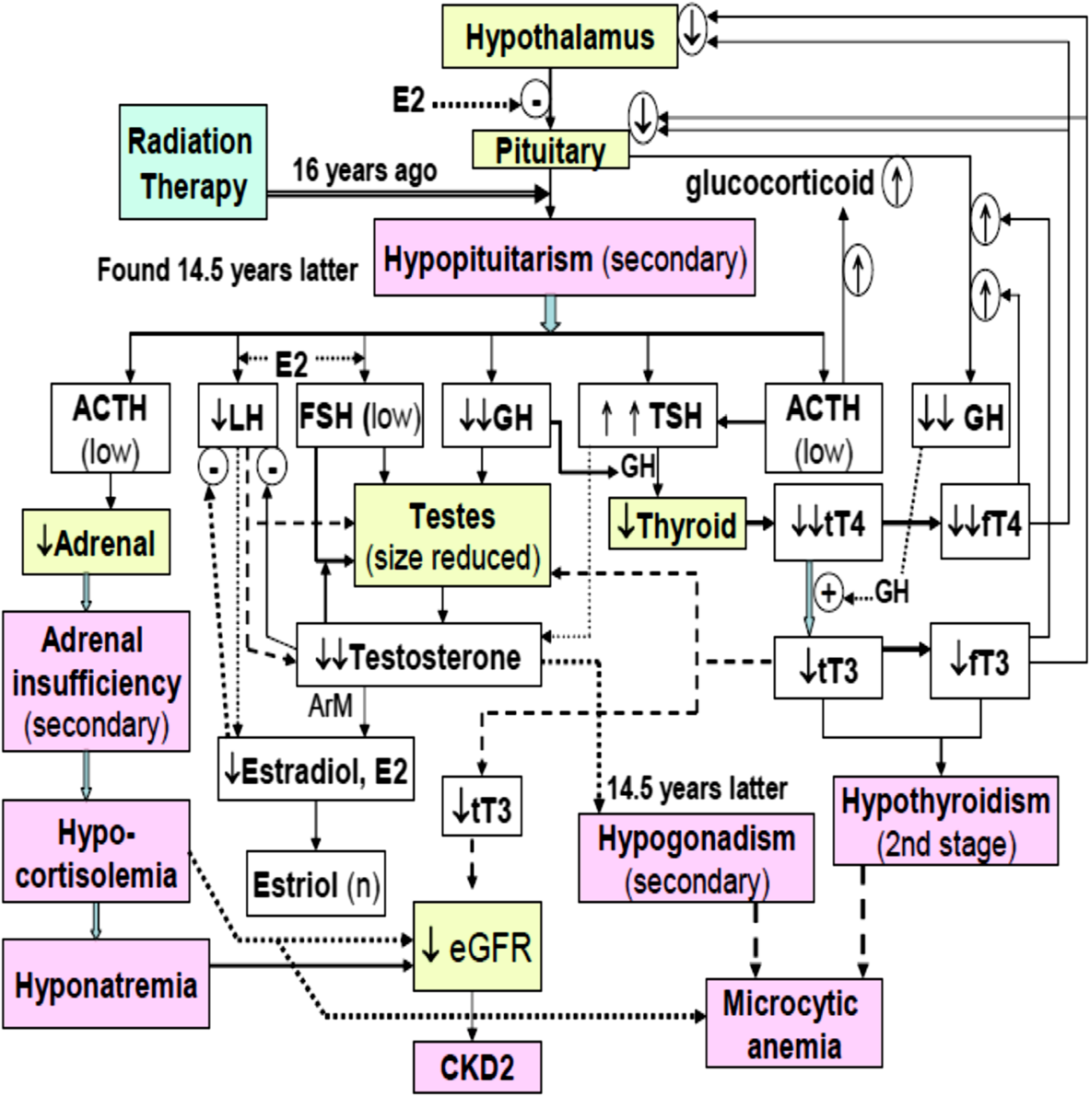
Schematic presentation indicating the pathological prevalence occurring in this male patient post radiotherapy for treating nasopharyngeal carcinoma (NPCA) 16 years ago (patient: male, age 38).

Concomitant occurrence of low end normal ACTH, extremely lowered HGH, and highly elevated TSH level with significantly lowered tT4 and fT4 (Table 1) implicated the second stage hypothyroidism (Figure 4). Buchanan et al. indicated that hypothyroidism also can be associated with increased FSH secretion [7]. However such a manifestation did not occur in this male victim (Table 1).

The HE stain of the patient’s seminiferous tubule revealed normal maturation of spermatozoa, but much less in population, implying infertility (Figure 3). Diminished secretion of LH can result in hypogonadism [8] (refer to Table 1 and Figure 4).

Secondary hypocortisolemia associated with hyponatremia (Table 3, Figure 4) occurred because of lacking adrenocorticotropin that is responsible for triggering the adrenal gland to produce the adrenal cortisol [9,10].

An e-GFR 89 mL/min/1.73 m^2^ implied stage 2 chronic kidney disease (CKD2) (Table 3). Hypocortisolemia (Table 1) can cause reduced e-GFR.

**Figure 5:**
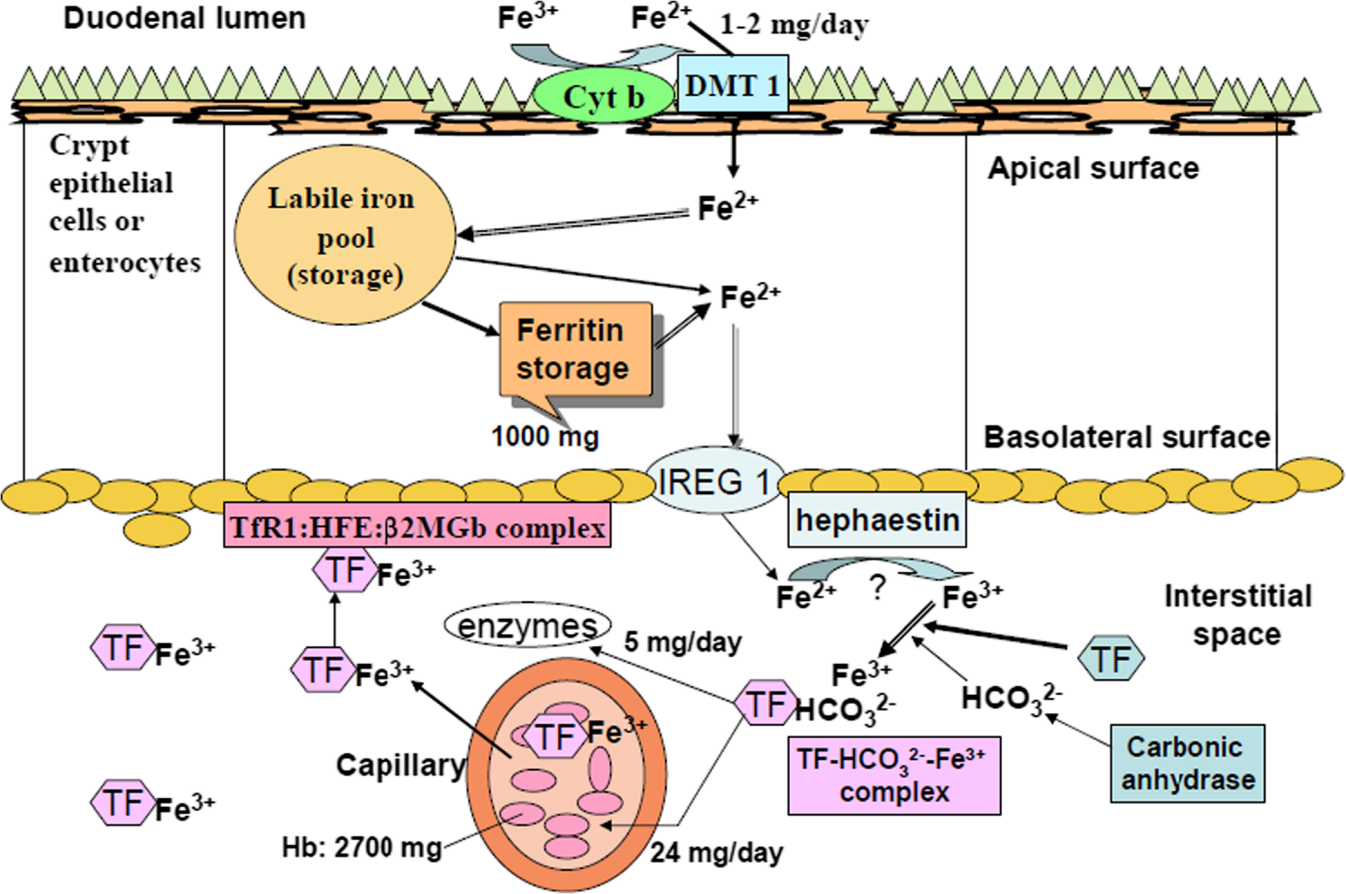
**The highly elevated serum transferrin due to lack of effective formation of ferric ion-carbonate-transferrin complex.** The dissociation constant of this complex is Kd =10^-22^[15]. Iron is absorbed from the duodenal lumen t by divalent-metal transporter 1 (DMT1) after reduced by the cytochrome b, Cyt-b. Intracellular iron enters the labile iron pool (LIP), either exported by iron-regulated protein 1 (IREG1) and then oxidized by hephaestin or, stored in ferritin cores [14] up to 1000 mg Fe. In the presence of bicarbonate ions supplied from anhydrase I, transferrin (Tf) and Fe^3+^ ions form a ternary complex ‘transferrin-bicarbonate-ferric ion complex’ to facilitate transport of ferric ions to be incorporated into hemoglobin [16,17]. A complex of transferrin receptor 1 (TfR1), the hemochromatosis protein (HFE) and β2-microglobulin, is supposed to act as primarily the iron-biosensor [14].

This patient was affiliated with microcytic anemia as evidenced by hypothyroidism (Table 1), low Hb, MCV (Table 2) and severely lowered ferritin (Table 3). Pituitary gland has an influence on erythropoiesis. Anaemia is thought to be due to loss of thyrotrophic and adrenotrophic hormones [11]. Testosterone enhances erythropoiesis by increasing renal erythropoietin production [11-13]. Severely lowered ferritin (Table 3) implies poor iron absorption by the duodenal lumen (Figure 5) [14].

This patient was commenced on replacement therapy with cortisone injection, Ringer’s solution (3%), thyroxine (T4), and testosterone replacement were immediately prescribed. Muscle strength was rapidly restored after replacement. Unlike primary hypogonadism, secondary hypogonadism often has a cause that is amenable to specific treatment. For that reason, finding the cause carries particular importance [18]. If the hypogonadism occurred postpubertally, usually only LH need to be replaced. In hypogonadism secondary to hypothalamic disease, spermatogenesis can also be stimulated by pulsatile administration of GnRH. Testosterone can be replaced whether the hypogonadism is primary or secondary. Unlike estrogen, testosterone itself is not suitable for oral replacement, because it is catabolized rapidly during its first pass through the liver. Derivatives of testosterone that are alkylated in the 17α position do not undergo this rapid hepatic catabolism; however, these agents appear to lack the full virilizing effect of testosterone, and they may cause hepatic toxicity, including cholestatic jaundice, a cystic condition of the liver called peliosis, and, possibly, hepatocellular carcinoma. Consequently, the 17α-alkylated androgens should not be used to treat testosterone deficiency [18].

During replacement therapy, clinicians should monitor patients for the efficacy and side effects of testosterone. Serum testosterone concentrations can vary with any of these preparations, so testosterone should be measured more than once to determine whether the initial dose is optimal. Serum testosterone should be measured again after a dose is changed and then once or twice a year. If the serum testosterone concentration is maintained within the normal range, the patient should experience reversal of the consequences of testosterone deficiency. Specifically, energy, libido, hemoglobin concentration, muscle mass, and bone density will increase [18]. Noteworthy, hypogonadism has been associated with several risk factors of atherosclerosis including obesity, Type II DM, dyslipidemia and hypertension.

Hypogonadism having low testosterone levels with increased carotid intima-media thickness (IMT) may evidence early atherosclerosis. In addition, low testosterone increases the susceptibility to myocardial ischemia. Erectile dysfunction is a symptom of hypogonadism, but also an end result of atherosclerosis and a predictor of coronary artery atherosclerosis (CAD) [19].

The replacement therapy was subsequently associated with rise in haemoglobin to 12.3 g/dL after 3 months and 13.5 g/dL, MCV 86 after 7 months. It is important to remember the role of pituitary hormones in erythropoiesis and consider hypopituitarism in the differential diagnosis of iron deficiency anaemia. Steroid and testosterone replacement corrected anaemia in this patient however it is not clear which hormone played a major role.

Hypopituitarism associated secondary adrenal insufficiency must be frequently noted to avoid the occurrence of severe hyponatremia. Replacement therapy with testosterone, thyroid hormone, coritsol and saline infusion are effective strategies. Cortisol inhibits sodium loss through the small intestine of mammals. Sodium depletion, however, does not affect cortisol levels. So cortisol cannot be used to regulate serum sodium [20]. The original task of cortisol may have been sodium transport [21].

Radiation-induced GHD may progressively and frequently develop in the first 10 years after radiation delivery [22]. Radiation induced anterior pituitary hormone deficiencies are irreversible and progressive; a recognized complication of cranial irradiation in cancer survivors–in particular, a very sensitivity and high incidence of GH deficiency (GHD) is observed [2], some cases may reach a prevalence of GHD between 50 and 100%. Replacement not only could improve the life quality, but also sustain the life expectancy.

Regular assessments of anterior-pituitary function are imperative in such patients, to achieve a timely diagnosis and to enable introduction of appropriate hormone-replacement therapy [2,23,24]. To increase tumour-related survival rates, a long-term monitoring tailored to the individual risk profile is required to avoid the sequelae of untreated pituitary hormonal deficiencies and resultant decrease in the quality of life [1].

## Conclusions

Damage resulting from radiotherapy is a progressive, chronic, and irreversible process. This case has taken 16 years to elicit PTX axis and adrenal dysfunction and concomitantly, secondary hypopituitarism complicated with second stage hypothyroidism, microcytic anemia, secondary hypocortisolemia with hyponatremia, secondary hypogonadism, and CKD2. Replacement therapy using T4, testosterone, cortisol, and 3% Ringer’s solution infusion has shown rather beneficial to his life quality.

To our believe, we are the first group who report such a complicate PTX dysfunction which also involves adrenal cortisol insufficiency, chronic kidney disease and microcytic anemia concomitantly in a single patient.

### Consent

Written informed consent was obtained from the patient for publication of this Case report and any accompanying images. A copy of the written consent is available for review by the Editor of this journal.

## Abbreviations

ACTH: Adrenocorticotropic hormone; LH: Leutenizing hormone; TSH: Thyroid stimulating hormone; HGH or GH: Human growth hormone; FSH: Follicular stimulating hormone; tT4: Total thyroxine; fT4: Free T4; tT3: Total T3; fT3: Free T3; E2: Estradiol; ArM: Aromatase; N: Normal.

## Competing interests

The authors declare that they have no competing interests.

## Authors’ contributions

YTL was responsible for concept, design, acquisition and interpretation of data. CCC and RYP contributed to design and critical revision of the manuscript and KCC was responsible for the critical revision of the manuscript. All authors have read and approved the final manuscript.

## Pre-publication history

The pre-publication history for this paper can be accessed here:

http://www.biomedcentral.com/1471-2490/14/19/prepub
